# Clinical and Radiographic Evaluation of the Effect of Platelet-Rich Fibrin (PRF) and NovaBone Bone Graft With and Without Decortication in Intrabony Defects: A Split-Mouth Clinical Study

**DOI:** 10.7759/cureus.84092

**Published:** 2025-05-14

**Authors:** Sarika P. S., Vidya Sagar S, Raja Babu, Jagadish Reddy Gooty

**Affiliations:** 1 Department of Periodontics and Implantology, Kamineni Institute of Dental Sciences, Nalgonda, IND; 2 Department of Periodontics, Kamineni Institute of Dental Sciences, Nalgonda, IND

**Keywords:** bone graft, clinical attachment level, decortication, intrabony defects, periodontal regeneration

## Abstract

Background: Intrabony defects resulting from chronic periodontitis can compromise oral health, necessitating regenerative interventions to restore lost periodontal structures. Using bone graft materials like NovaBone and biologics like platelet-rich fibrin (PRF) has shown favorable outcomes. Decortication, a technique that enhances angiogenesis and graft stability, may improve regeneration. This study aimed to assess the clinical and radiographic outcomes of PRF combined with NovaBone putty, with and without decortication, in managing intrabony defects.

Methodology: A randomized controlled clinical study was conducted on 15 patients with 30 intrabony defect sites. Subjects were divided into two groups: Group I received PRF and NovaBone without decortication, while Group II received the same combination with decortication. Clinical parameters, including plaque index, gingival index, gingival bleeding index (GBI), probing pocket depth (PPD), clinical attachment level (CAL), and gingival recession (GR), along with radiographic bone fill, were evaluated at baseline, three months, and six months.

Results: Both groups demonstrated significant improvements in PPD reduction, CAL gain, and bone fill over time (p < 0.05). Group II showed a greater reduction in GBI (93.33%) compared to Group I (80%). Mean CAL gain was 2.66 mm in Group I and 3.04 mm in Group II. Radiographic assessment revealed greater bone fill in Group II (27.26%) than Group I (20.93%). Changes in GR were minimal and statistically insignificant.

Conclusion: Combining PRF with NovaBone yields positive regenerative outcomes in intrabony defects, and incorporating decortication offers additional clinical benefits.

## Introduction

Periodontal disease, a leading cause of tooth loss globally, significantly diminishes an individual's quality of life [1]. Chronic periodontitis is a prevalent inflammatory condition affecting the supporting structures of the teeth, resulting in attachment loss, bone destruction, pocket formation, and gingival recession (GR). Although primarily observed in adults, it can affect individuals of any age and is closely associated with plaque and calculus accumulation [2].

Intrabony defects develop due to the apical migration of plaque, contributing to disease progression when left untreated. These defects may be managed using either resective or regenerative techniques, with regeneration being the preferred approach. Periodontal regeneration aims to restore the lost periodontal tissues, including the alveolar bone, periodontal ligament, and cementum [3]. Among various regenerative strategies, bone grafting remains a widely accepted modality, utilizing materials such as autografts, allografts, xenografts, and alloplasts. Bioactive glass, a type of alloplast, has shown promising results due to its osteoconductive and osteostimulative properties. It promotes bone formation while preventing epithelial downgrowth and undesirable fibroblast proliferation.

Platelet-rich fibrin (PRF), a second-generation platelet concentrate, has gained attention for its wound-healing properties. It consists of a fibrin matrix enriched with platelets, leukocytes, and growth factors such as platelet-derived growth factor (PDGF) and transforming growth factor (TGF)-β, which support angiogenesis, cell migration, and proliferation [4].

Decortication, or intramarrow penetration (IMP), is a surgical technique involving perforation of the cortical bone to enhance vascularization and facilitate the recruitment of progenitor cells. This process may promote better integration of the bone graft. However, its clinical effectiveness in treating intrabony defects remains inconclusive and warrants further investigation [5,6]. Therefore, the study aimed to evaluate and compare the clinical and radiographic outcomes of PRF and NovaBone putty, with and without decortication, in treating intrabony periodontal defects.

## Materials and methods

**Figure 1:**
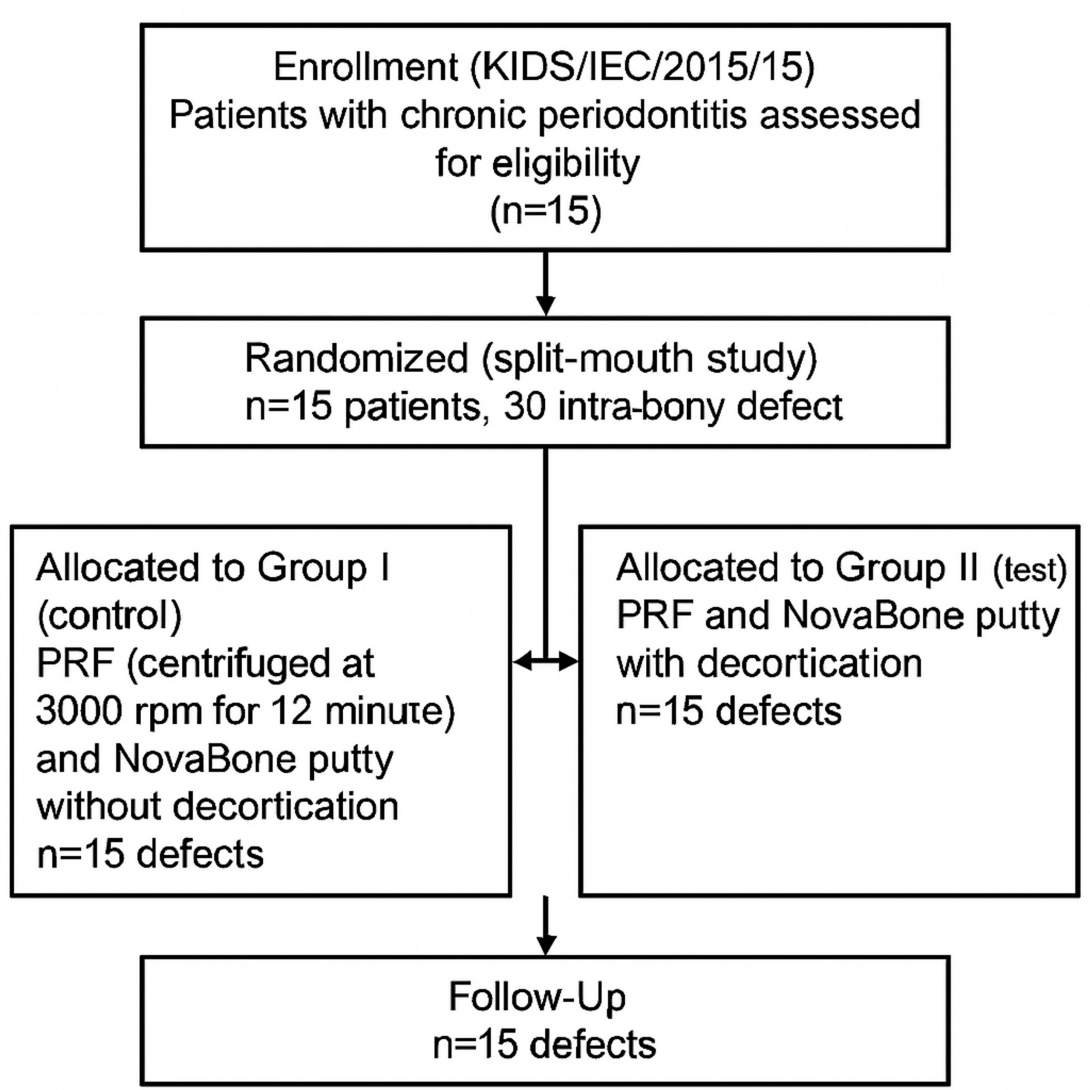
Study protocol KIDS: Kamineni Institute of Dental Sciences; IEC: Institutional Ethics Committee; PRF: platelet-rich fibrin

After obtaining institutional ethical approval (KIDS/IEC/2015/15), patients were recruited for a randomized controlled clinical study conducted in the Department of Periodontics, assessing the impact of decortication as an adjunct to PRF and NovaBone putty in managing intrabony defects. Fifteen patients diagnosed with chronic periodontitis were enrolled, with a total of 30 intrabony defects confirmed through clinical and radiographic assessments (Figure 1). Participants were aged 28 years or older and presented with a probing pocket depth (PPD) of ≥5 mm and V-shaped defects identified on intraoral periapical radiographs. Exclusion criteria included pregnancy, lactation, systemic illness, smoking, history of periodontal treatment or antibiotic therapy in the past six months, and current medication use. Detailed medical and dental histories were recorded, and informed consent was obtained from all participants.

**Figure 2:**
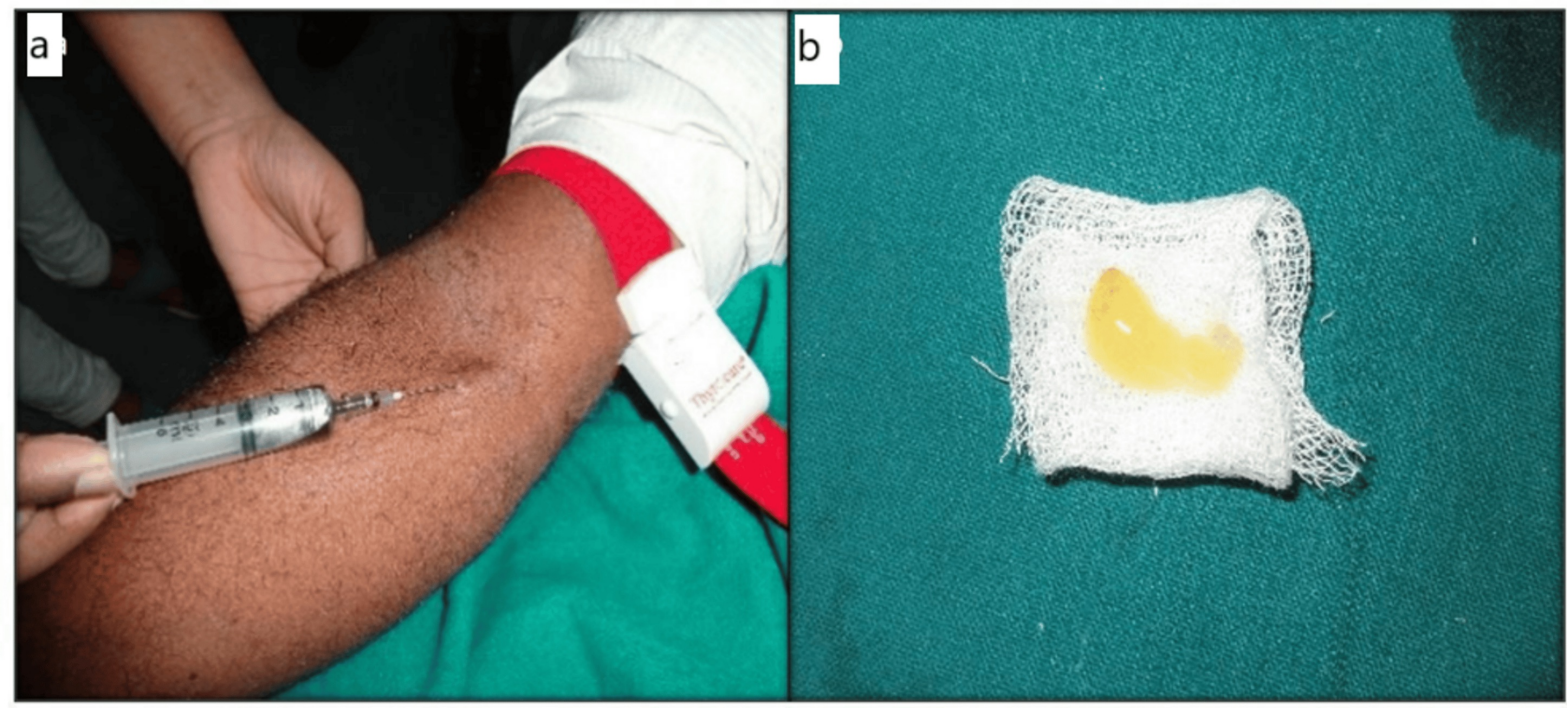
Control and test group PRF preparation. (a) Venipuncture performed on patient. (b) PRF membrane prepared PRF: platelet-rich fibrin

Six weeks after scaling and root planing, the 30 defects were randomly assigned to two treatment groups. Group I (control) received PRF (centrifuged at 3,000 rpm for 12 minutes) and NovaBone putty without decortication, while Group II (test) received PRF and NovaBone putty with decortication. NovaBone bone graft material (NovaBone Products, LLC, USA) was used in all cases. PRF was prepared by drawing 10 mL of venous blood into tubes without anticoagulant (Figure 2a), followed by immediate centrifugation at 3,000 rpm for 12 minutes to yield three layers. The middle fibrin clot was isolated and placed on sterile gauze (Figure 2b).

Clinical parameters recorded at baseline, three months, and six months included plaque index (PI), gingival index (GI), gingival bleeding index (GBI), PPD, clinical attachment level (CAL), GR, and radiographic bone fill. The PI was measured at four sites per tooth with scores ranging from 0 (no plaque) to 3 (abundant plaque), categorizing oral hygiene from excellent (0) to poor (2.0-3.0). GI scores ranged from 0 (healthy gingiva) to 3 (severe inflammation with spontaneous bleeding). GBI assessed bleeding on probing and was recorded as present (1) or absent (0). PPD and CAL were evaluated using a University of North Carolina-15 probe, measuring from the gingival margin and cementoenamel junction (CEJ), respectively, to the base of the pocket. To ensure reproducibility and minimize measurement variability, PPD and CAL were assessed with the aid of a customized acrylic stent that provided a fixed reference point and standardized probe angulation. This facilitated accurate longitudinal comparisons at each time point. GR was measured from the CEJ to the gingival margin. Radiographic assessment used standardized radiovisiography and image analysis software (University of Texas Health Science Center at San Antonio) to measure the vertical distance from the alveolar crest to the base of the defect.

Surgical procedures for both groups were carried out under strict aseptic conditions following administration of local anesthesia using 2% lignocaine with 1:80,000 adrenaline. A crevicular incision was made using a No. 15 Bard-Parker blade (Bard-Parker® Stainless Steel, Aspen Surgical, Caledonia, UA), and full-thickness mucoperiosteal flaps were carefully elevated to provide adequate access to the defect site, while maintaining the integrity of the interdental papilla wherever possible. The defect sites were thoroughly debrided and degranulated using Gracey curettes (Hu-Friedy, Frankfurt, Germany) to ensure complete removal of granulation tissue and allow for optimal graft adaptation.

**Figure 3:**
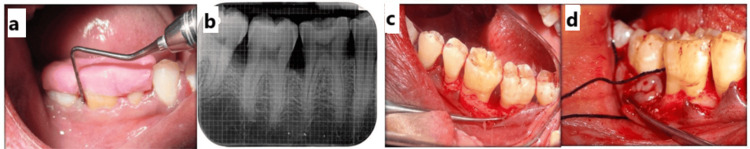
Control group surgical procedure with postoperative three- and six-month radiographs. (a) Measurement of PPD using a stent. (b) Baseline radiograph. (c) Flap elevation showing an intrabony defect distal to 46. (d) NovaBone putty, PRF membrane place PPD: probing pocket depth

**Figure 4:**
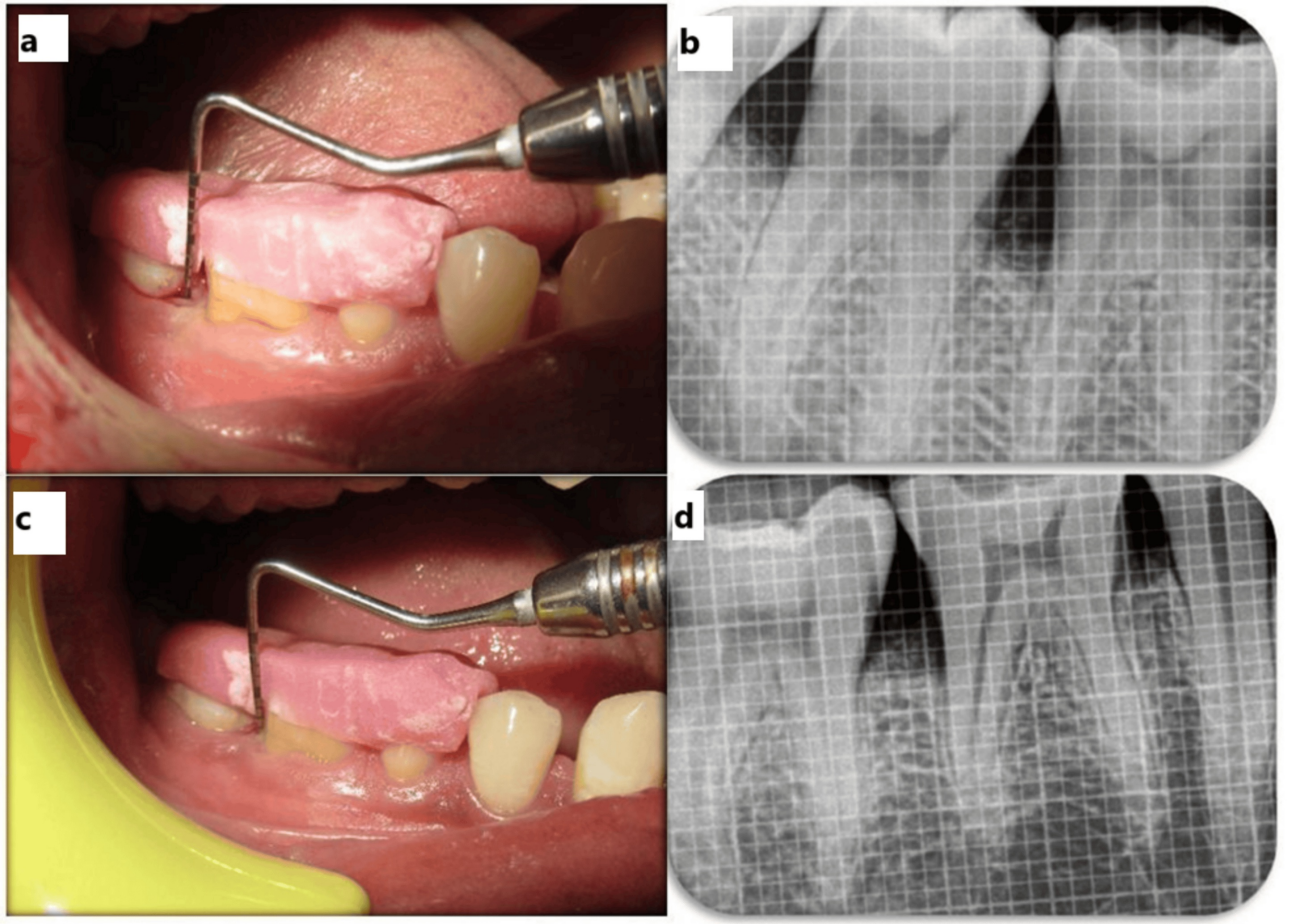
Control group postoperative follow-up. (a,b) Three-month clinical and radiographic follow-up. (c,d) Six-month clinical and radiographic follow-up

In Group I, the defect was treated using a combination of autologous PRF and NovaBone putty. PRF was prepared using standard centrifugation protocols and placed into the defect along with NovaBone, a synthetic bioactive graft. The flap was repositioned and sutured with interrupted stitches, and a periodontal dressing (Coe-Pak, GC International AG, Luzern, Switzerland) was applied to protect the site (Figure 3). Follow-up clinical (Figures 4a, 4c) and radiographic (Figures 4b, 4d) evaluations were performed at three and six months.

**Figure 5:**
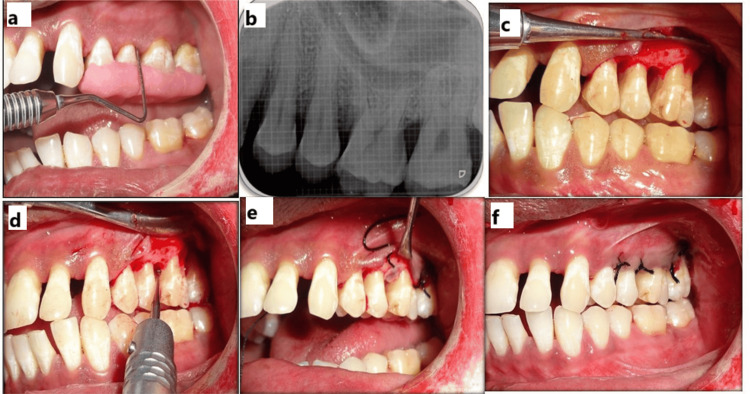
Test group. (a) Measurement of PPD using a stent. (b) Baseline radiograph. (c) Flap elevation showing an intrabony defect distal to 25. (d) Decortication in relation to the distal end of 25. (e) NovaBone putty, PRF membrane placed. (f) Sutures given PPD: probing pocket depth; PRF: platelet-rich fibrin

**Figure 6:**
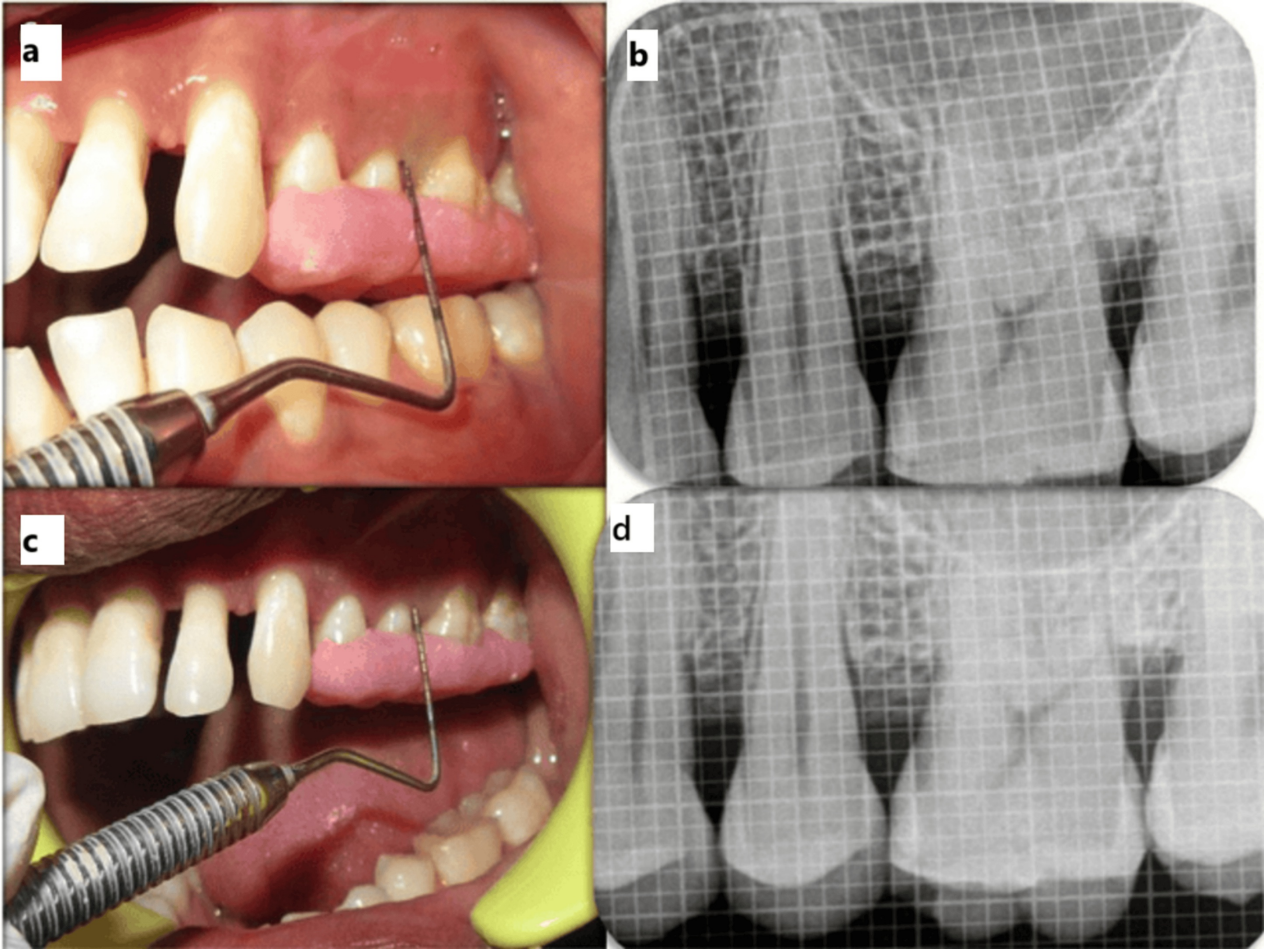
Test group postoperative follow-up. (a,b) Three-month clinical and radiographic follow-up. (c,d) Six-month clinical and radiographic follow-up

In Group II, in addition to the Group I protocol, decortication was performed following flap elevation and debridement. Small perforations were made in the cortical bone using a round bur under saline irrigation to promote bleeding and facilitate the release of marrow-derived progenitor cells and growth factors. This is intended to enhance angiogenesis and bone regeneration. PRF and NovaBone were then placed in the defect, the site was sutured, and a periodontal dressing was applied (Figures 5a-5f). Standard postoperative care was provided. Clinical (Figures 6a, 6c) and radiographic (Figures 6b, 6d) evaluations were carried out at three and six months to assess healing outcomes.

Postoperative care was standardized for both groups and included a seven-day regimen of amoxicillin + cloxacillin (500 mg three times a day), along with diclofenac sodium (50 mg twice a day) for the first three days to manage pain and inflammation. Antiseptic care was reinforced using 0.2% chlorhexidine mouth rinse twice daily for two weeks. Patients received comprehensive verbal and written postoperative instructions. Sutures and dressing were removed on the seventh postoperative day, and the surgical site was irrigated with normal saline and 3% hydrogen peroxide to ensure cleanliness and facilitate healing. At each follow-up, reinforcement of oral hygiene practices was provided, and supragingival scaling was performed if necessary. Clinical and radiographic assessments were repeated at each interval to evaluate periodontal healing and the regenerative outcome of the interventions.

Statistical analysis was performed using Statistical Package for the Social Sciences version 20.0 (SPSS Inc., Chicago, IL). The normality of data distribution was confirmed via the Kolmogorov-Smirnov test. Intergroup comparisons were conducted using the independent t-test, while intragroup changes at different time intervals were analyzed using the paired t-test. A p-value of <0.05 was considered statistically significant.

## Results

A total of 30 defect sites (n = 30) were evaluated, 15 each in the control and test groups. All subjects completed the six-month follow-up period. Data are presented as mean ± standard deviation and were analyzed using paired and unpaired t-tests or Wilcoxon signed-rank tests, as appropriate.

Table 1 compares the GBI values within the control and test groups over time. In the control group, GBI decreased significantly from 1.00 at baseline to 0.53 at three months (46.67% reduction, p = 0.0180) and to 0.20 at six months (80.00% reduction, p = 0.0022). The test group showed a more pronounced reduction, from 1.00 at baseline to 0.40 at three months (60.00%, p = 0.0077) and 0.07 at six months (93.33%, p = 0.0010). Further significant decreases were noted between three and six months in both groups. The Wilcoxon signed-rank test was used for statistical analysis, indicating significant within-group improvements, with the test group showing greater efficacy.

**Table 1:** Wilcoxon signed-rank test used for intra- and intergroup comparisons of gingival bleeding index at different time intervals ^*^Statistically significant (p < 0.05) SD: standard deviation

Group	Time comparison	n	Mean difference ± SD	% Change	Z value	p value^*^
Control	Baseline vs. three months	15	0.47 ± 0.52	46.67%	2.3661	0.0180
	Baseline vs. six months	15	0.80 ± 0.41	80.00%	3.0592	0.0022
	Three months vs. six months	15	0.33 ± 0.49	62.50%	2.0223	0.0431
Test	Baseline vs. three months	15	0.60 ± 0.51	60.00%	2.6656	0.0077
	Baseline vs. six months	15	0.93 ± 0.26	93.33%	3.2958	0.0010
	Three months vs. six months	15	0.33 ± 0.49	83.33%	2.0226	0.0431

Table 2 shows intergroup comparisons of PPD between the control and test groups at baseline, three months, and six months using the independent t-test. At baseline, three months, and six months, no significant differences were found between the groups (p > 0.05), indicating that both interventions had similar effects. Additionally, comparisons between time points within each group (baseline vs. three months, baseline vs. six months, three months vs. six months) showed no significant changes over time (p > 0.05), suggesting that both groups maintained comparable results throughout the study period.

**Table 2:** Independent t-test used for intergroup comparison of probing pocket depth at baseline, three months, and six months BL: baseline; 3M: three months; 6M: six months; SD: standard deviation

Time comparison	Group	n	Mean ± SD	t value	p value
Baseline	Control group	15	7.53 ± 1.25	0.3044	0.7631
	Test group	15	7.73 ± 2.22		
Three months	Control group	15	4.73 ± 1.16	-0.4337	0.6679
	Test group	15	4.53 ± 1.36		
Six months	Control group	15	3.67 ± 1.11	-1.4856	0.1486
	Test group	15	3.13 ± 0.83		
BL-3M	Control group	15	2.80 ± 1.26	0.7315	0.4706
	Test group	15	3.20 ± 1.70		
BL-6M	Control group	15	3.87 ± 1.41	1.0772	0.2906
	Test group	15	4.60 ± 2.23		
3M-6M	Control group	15	1.07 ± 0.88	0.9378	0.3564
	Test group	15	1.40 ± 1.06		

Table 3 shows a significant reduction in PPD within both control and test groups over six months (p < 0.05). In the control group, PPD reduced from 7.53 mm at baseline to 3.67 mm at six months, a 51.33% reduction. In the test group, PPD decreased from 7.73 to 3.13 mm, showing a greater 59.48% reduction. Statistically significant improvements were observed at all intervals within both groups, indicating effective periodontal healing over time.

**Table 3:** Paired t-test used for intragroup comparison of PPD in control and test groups across time intervals ^*^Statistically significant (p < 0.05) SD: standard deviation

Group	Time	n	Mean ± SD	% of change	Paired t	p value
Control	Baseline	15	7.53 ± 1.25	-	-	-
	Three months	15	4.73 ± 1.16	37.17	8.5732	0.0001^*^
	Six months	15	3.67 ± 1.11	51.33	10.6401	0.0001^*^
	Three to six months	15	3.67 ± 1.11	22.54	4.6748	0.0004^*^
Test	Baseline	15	7.73 ± 2.22	-	-	-
	Three months	15	4.53 ± 1.36	41.38	7.2957	0.0001^*^
	Six months	15	3.13 ± 0.83	59.48	7.9903	0.0001^*^
	Three to six months	15	3.13 ± 0.83	30.88	5.1366	0.0002^*^

A paired t-test applied for intragroup comparison revealed a statistically significant gain in clinical attachment level (CAL) in both control and test groups over the six months. The control group showed a CAL gain of 2.67 mm at three months and 3.73 mm at six months from baseline, with percentage improvements of 30.53% and 42.75%, respectively (p < 0.0001). The test group demonstrated greater CAL gains of 3.07 mm at three months and 4.40 mm at six months, reflecting improvements of 33.82% and 48.53% from the baseline (p < 0.0002 and p < 0.0001, respectively). Additionally, significant improvements were also observed between the three- and six-month intervals within both groups, indicating continuous periodontal healing over time (Table 4).

**Table 4:** Paired t-test applied for intragroup comparison of clinical attachment level ^*^Statistically significant (p < 0.05) SD: standard deviation

Group	Time	n	Mean ± SD (mm)	% of change	Paired t	p value
Control	Baseline	15	8.73 ± 2.19	-	-	-
	Three months	15	6.07 ± 1.83	30.53%	6.3246	0.0001^*^
	Six months	15	5.00 ± 1.96	42.75%	9.4273	0.0001^*^
	Three vs. six months	15	-	17.58%	4.6748	0.0004^*^
Test	Baseline	15	9.07 ± 2.89	-	-	-
	Three months	15	6.00 ± 2.10	33.82%	5.0666	0.0002^*^
	Six months	15	4.67 ± 1.91	48.53%	5.8304	0.0001^*^
	Three vs. six months	15	-	22.22%	4.3944	0.0006^*^

Intragroup comparison of GR scores was conducted using the paired t-test (n = 15 per group). A marginal increase in GR was observed in both the control group (from 1.60 ± 1.84 to 1.67 ± 1.45 mm) and the test group (from 1.47 ± 1.30 to 1.73 ± 1.44 mm) over six months. However, these changes were not statistically significant at any time interval (p > 0.05), indicating that neither treatment approach led to a meaningful change in GR (Table 5).

**Table 5:** Paired t-test used for intragroup comparison of gingival recession scores No statistically significant differences observed (p  >  0.05) SD: standard deviation

Group	Time	n	Mean ± SD (mm)	% of change	Paired t	p value
Control	Baseline	15	1.60 ± 1.84	-	-	-
	Three months	15	1.67 ± 1.40	-4.17%	-0.3669	0.7192
	Six months	15	1.67 ± 1.45	-4.17%	-0.3232	0.7513
	Three vs. six months	15	-	0.00%	0.0000	1.0000
Test	Baseline	15	1.47 ± 1.30	-	-	-
	Three months	15	1.67 ± 1.35	-13.64%	-0.8231	0.4243
	Six months	15	1.73 ± 1.44	-18.18%	-1.0745	0.3008
	Three vs. six months	15	-	-4.00%	-1.0000	0.3343

The paired t-test was applied to assess the intragroup differences in radiographic defect fill between baseline, three months, and six months for both control and test groups. In the control group, a significant reduction in the defect fill was observed from baseline (7.69 ± 1.73 mm) to six months (6.08 ± 1.59 mm), with a mean difference of 1.61 mm (20.93% change, p < 0.0001). A similar trend was seen in the test group, where the defect fill decreased from baseline (7.95 ± 2.68 mm) to six months (5.78 ± 2.15 mm), with a mean difference of 2.17 mm (27.26% change, p < 0.0001). Statistically significant improvements were noted at all time intervals (baseline to three months, baseline to six months, and three to six months) in both groups (p < 0.05), indicating effective radiographic defect resolution over time (Table 6).

**Table 6:** Paired t-test applied for intragroup comparison of radiographic defect fill in control and test groups ^*^Statistically significant (p  <  0.05) SD: standard deviation

Group	Time	n	Mean ± SD (mm)	% of change	Paired t	p value
Control	Baseline	15	7.69 ± 1.73	-	-	-
	Three months	15	6.75 ± 1.91	12.22%	6.1349	0.0001^*^
	Six months	15	6.08 ± 1.59	20.93%	9.2420	0.0001^*^
	Three vs. six months	15	^-^	9.92%	4.5411	0.0005^*^
Test	Baseline	15	7.95 ± 2.68	-	-	-
	Three months	15	6.64 ± 2.22	16.52%	6.1188	0.0001^*^
	Six months	15	5.78 ± 2.15	27.26%	9.9980	0.0001^*^
	Three vs. six months	15	-	12.87%	6.8803	0.0001^*^

## Discussion

Untreated periodontal disease leads to tooth loss by damaging supporting structures. Periodontal therapy aims to halt disease progression and regenerate lost tissues, with bone grafting being a key approach. While autogenous grafts are effective, their use is often impractical, leading to the adoption of synthetic and natural bone substitutes for regenerative treatments in intrabony defects, ridge augmentation, socket preservation, and sinus floor elevation [7]. An ideal synthetic graft should support osteointegration, osteoconduction, and mimic natural bone mechanics to prevent stress shielding and fractures [8]. Bioactive glass, a common alloplast, has limitations as a scaffold, which are addressed by calcium phosphosilicate (CPS) putty like NovaBone. CPS putty offers osteostimulative and osteoconductive benefits, promotes clot stabilization, enhances healing, and is easy to handle [9]. Studies by Froum et al. [10] and Lovelace et al. [11] have shown superior clinical and radiographic outcomes with bioactive glass in periodontal defects, with CPS putty demonstrating high bone regeneration potential compared to glass ceramics and hydroxyapatite.

Xynos et al. [12] reported that the ionic products of glass dissolution influence gene expression, upregulating key genes involved in cell cycle regulation, growth, and apoptosis, including insulin-like growth factor (IGF)-II, a potent osteoblast mitogenic factor. These osteostimulative properties have established CPS as an effective material for periodontal defect treatment. Studies by Trombelli et al. [13] and Agrawal et al. [14] have demonstrated significant improvements in periodontal indices over six months in bioactive glass-treated sites. Growth factors have also shown promise in periodontal regeneration by regulating cell proliferation, differentiation, and matrix synthesis. Choukroun’s PRF, a second-generation platelet concentrate, releases growth factors such as PDGF, TGF, vascular endothelial growth factor (VEGF), epidermal growth factor, and IGF-1, promoting tissue regeneration [15]. PRF forms a fibrin matrix with platelets, leukocytes, cytokines, and circulating stem cells, allowing sustained release of growth factors like TGF-β1, PDGF-AB, and VEGF. In this study, PRF-treated groups showed clinical and radiographic improvements, aligning with findings from Sharma and Pradeep [16] and Park et al. [17].

Decortication of bone before graft placement is a key component of guided bone regeneration (GBR), facilitating vascularization and progenitor cell migration to the treated site. It mimics natural bone remodeling by creating perforations that enhance capillary ingrowth, which is crucial for bone deposition. Since osteoblasts originate from the periosteum, endosteum, and bone marrow, elevating the periosteum during GBR may limit their contribution [18]. Decortication helps overcome this by allowing faster access for osteoblasts, blood vessels, and pluripotent cells to the grafted site [19]. Studies suggest that decortication using a round bur improves bone healing by enhancing local vascular supply, clot formation, and stabilization [20]. IMP has been clinically utilized as an adjunct in bone grafting, GBR, and open flap debridement procedures, with several studies reporting enhanced bone fill [21]. However, findings by Sepe et al. did not demonstrate a statistically significant difference, highlighting the need for further investigation into the efficacy of IMP [22]. Crea et al. highlighted the benefits of IMP in periodontal procedures, showing significant CAL gain and radiographic defect fill over six months [23]. This study compared PRF and NovaBone putty with and without decortication in treating intrabony defects, showing clinical and radiographic improvements in both groups, with better outcomes in the decortication group (PRF + NovaBone putty).

Clinical outcomes assessed included PI, GBI, GI, probing depth (PD), CAL, and GR, while radiographic analysis focused on CEJ bottom of a defect measurements. Plaque and GI scores showed a statistically significant reduction over six months (p < 0.05), indicating patient compliance with oral hygiene. GBI scores also decreased significantly in both groups (p < 0.05), aligning with Thorat et al., who found PRF-based treatments reduced GBI but showed no intergroup differences [24]. Similarly, Park et al. reported a significant reduction in gingival bleeding, reflecting decreased postsurgical inflammation [25]. GR increased slightly (0.20-0.5 mm) at three and six months, consistent with Hou et al., who observed a 0.3-0.8 mm increase after surgery with decortication [26]. PD reduction and CAL gain remain the primary measures of periodontal regeneration, ensuring long-term maintenance. Though histological evaluation is the gold standard, improvements in PD and CAL serve as practical indicators of successful regenerative outcomes.

The present study demonstrated a significant reduction in PD and CAL gain in both groups from baseline to six months, aligning with findings from Trombelli et al. [27] and Hench and Paschall [28]. The use of PRF with NovaBone putty showed promising regenerative potential, supporting previous research on bioactive glass as a scaffold for growth factor delivery. Radiographic analysis confirmed defect fill comparable to studies by Lakshmi et al. [29], indicating minimal crestal resorption. PRF’s sustained release of growth factors, as observed by He et al. [30], contributes to improved periodontal regeneration by enhancing osteoblast activity and soft tissue healing.

In the present study, PPD and CAL measurements were performed using a UNC-15 probe with the aid of a customized acrylic stent to enhance measurement accuracy. The use of a stent provided a fixed reference point and ensured consistent probe angulation across all time intervals, thereby reducing intra- and interexaminer variabilities. Accurate and reproducible measurement of periodontal parameters is essential for evaluating treatment outcomes, especially in longitudinal studies. The importance of using a stent during probing has been well-documented in the literature. Fernandes and Muller [31] emphasized that incorporating stents into periodontal probing protocols facilitates standardization and minimizes technique-sensitive errors, ultimately improving the reliability of clinical assessments. Our findings support this recommendation, as the use of a stent contributed to consistent and precise documentation of clinical improvements over the six-month follow-up period.

In the present study, a split-mouth randomized controlled trial design was chosen to minimize interindividual variability in factors such as oral hygiene practices, immune response, and systemic health, which can significantly influence periodontal treatment outcomes. This approach allows for direct intraindividual comparison between test and control sites, thereby enhancing the internal validity of the study. Our design is consistent with the methodology employed by Skumar et al. [32], who conducted a split-mouth randomized controlled trial to evaluate the regenerative potential of PRF combined with bioactive glass in treating horizontal bone defects in Stage II and III periodontitis. Their findings demonstrated that the split-mouth approach provided reliable and standardized comparisons between treatment modalities by controlling for confounding variables within the same subject. Similarly, in our study, utilizing this design enabled us to evaluate the adjunctive effect of decortication on PRF and NovaBone putty under uniform systemic and local conditions, thereby improving the accuracy and strength of our comparative analysis.

However, limitations include reliance on clinical and radiographic assessments without histological validation, a short follow-up duration of six months, and variability in defect morphology and sample size. Further long-term studies with larger cohorts and histological analysis are recommended to validate these findings.

## Conclusions

Within the limitations of this study, both the control (PRF + NovaBone Putty) and test groups (Decortication + PRF + NovaBone Putty) demonstrated significant clinical and radiographic improvements over six months. The test group showed greater reductions in PPD and greater CAL gain, along with more substantial radiographic bone fill, indicating that decortication enhances periodontal regeneration. These findings support the adjunctive use of decortication in regenerative periodontal procedures, warranting further investigation through long-term, multicentric trials with histological validation.
